# Supplementary material for: Integrated virtual screening and MD simulation study to discover potential inhibitors of mycobacterial electron transfer flavoprotein oxidoreductase
Source: PLoS One. 2024 Nov 15;19(11):e0312860. doi: 10.1371/journal.pone.0312860 (PMC11567552; doi:10.1371/journal.pone.0312860)

S1 Fig. flDPnn and predicted aligned error (PAE). (a) flDPnn (putative function- and linker-based Disorder Prediction using deep neural network). (b) PAE 2D heatmap.

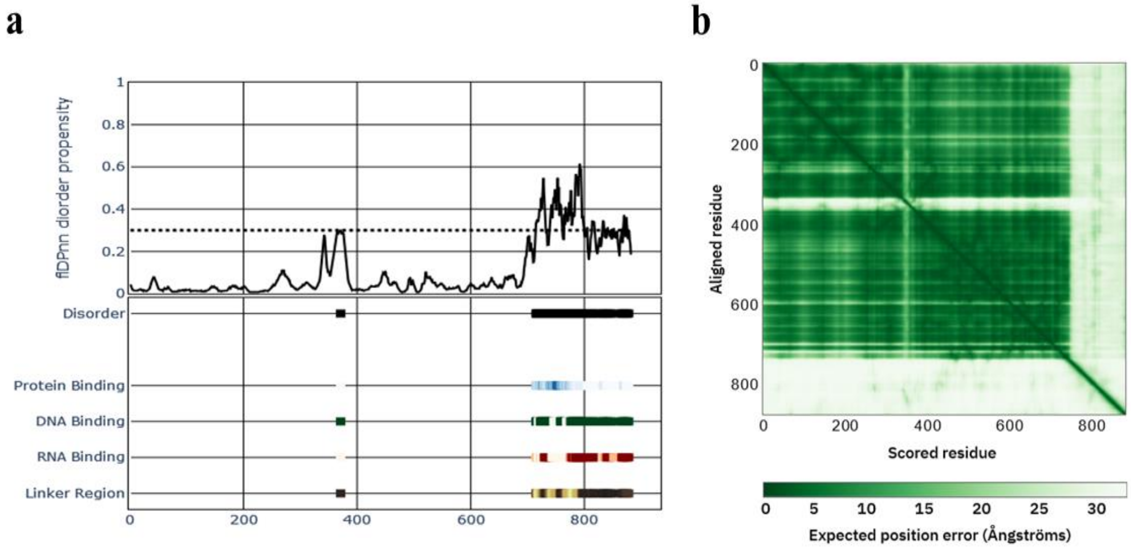

Supplement: S1 Fig — (a) flDPnn (putative function- and linker-based Disorder Prediction using deep neural network). (b) PAE 2D heatmap. (PDF) [file pone.0312860.s001.pdf]
