# Supplementary figures and images for: Integrated virtual screening and MD simulation study to discover potential inhibitors of mycobacterial electron transfer flavoprotein oxidoreductase
Source: PLoS One. 2024 Nov 15;19(11):e0312860. doi: 10.1371/journal.pone.0312860 (PMC11567552; doi:10.1371/journal.pone.0312860)

**S2 Fig. MolProbity Ramachandran plot.**

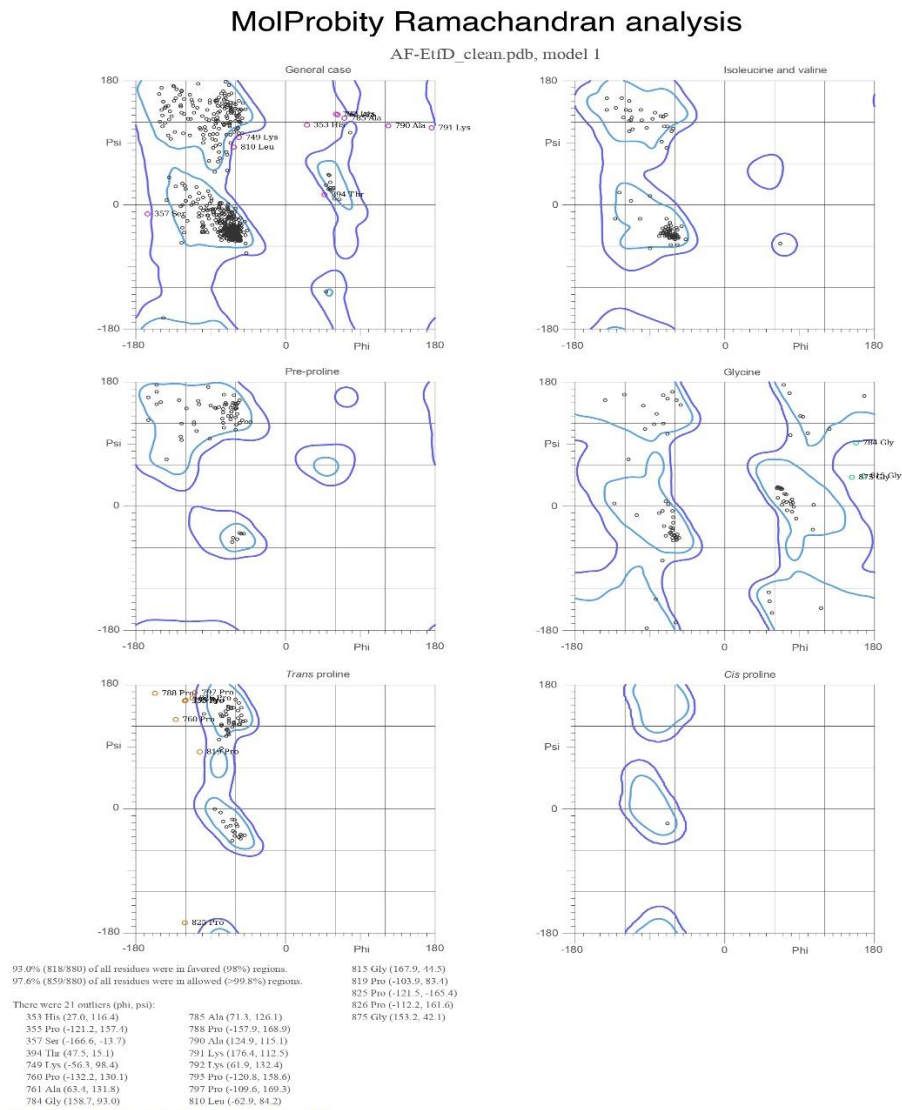

<http://kinemage.biochem.duke.edu>

Lovell, Davis, et al. Proteins 50:437 (2003)

Supplement: S2 Fig — (PDF) [file pone.0312860.s002.pdf]

S3 Fig. ProSA Z-score.

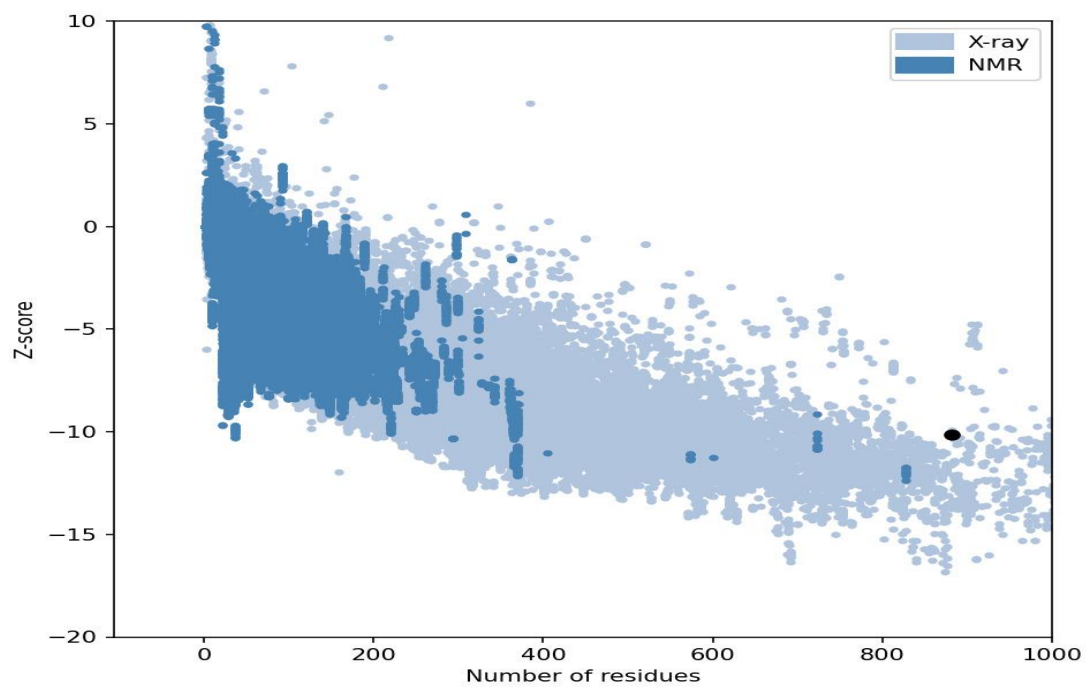

Supplement: S3 Fig — (PDF) [file pone.0312860.s003.pdf]

S4 Fig. ProSA residue energies.

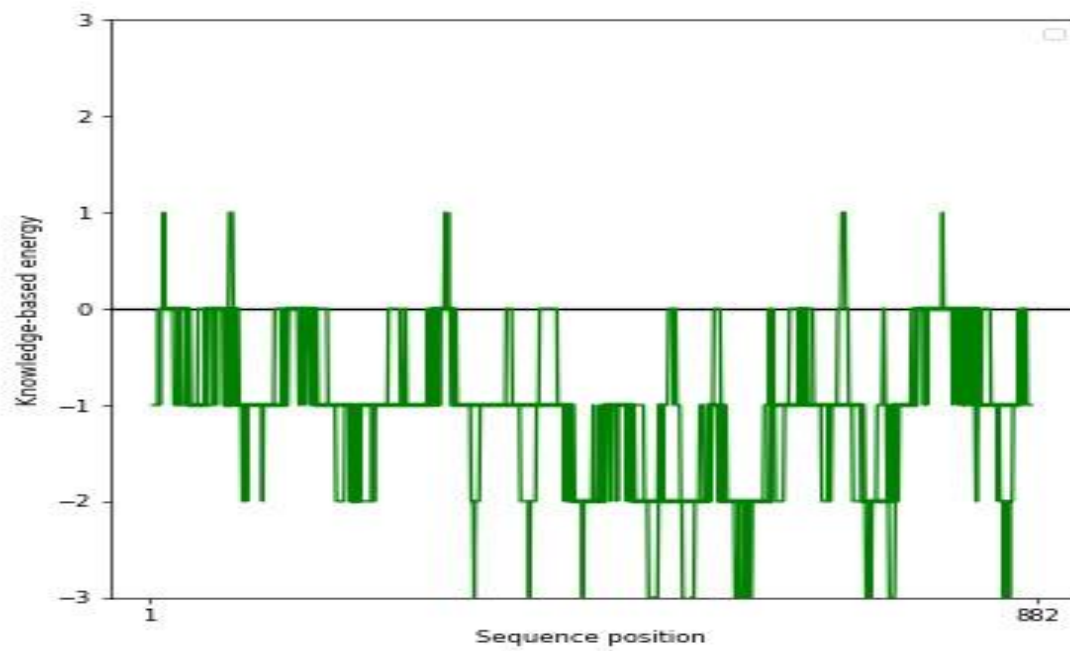

Supplement: S4 Fig — (PDF) [file pone.0312860.s004.pdf]

S6 Fig. Molecular docking 2D representations.

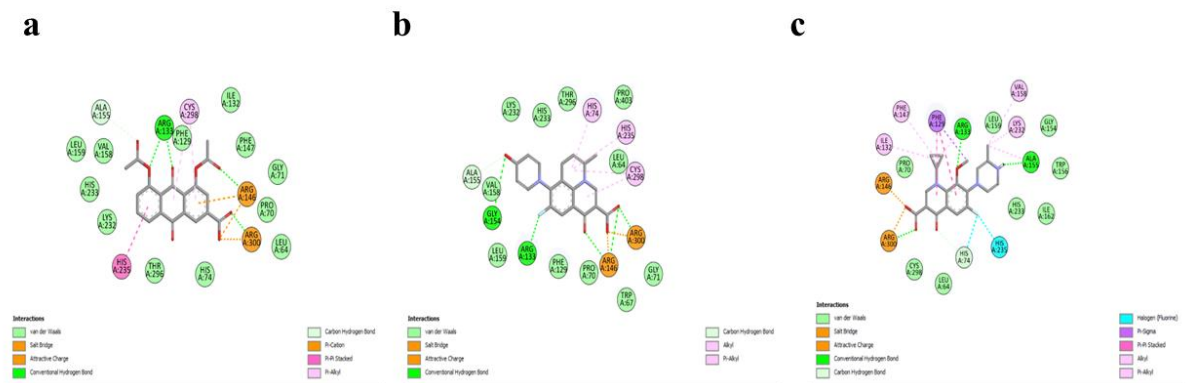

Supplement: S6 Fig — (PDF) [file pone.0312860.s006.pdf]

color scheme follows that of Fig 3d.

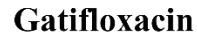

Supplement: S7 Fig — The color scheme follows that of Fig 3d. (PDF) [file pone.0312860.s007.pdf]
