# Supplementary material for: Integrated virtual screening and MD simulation study to discover potential inhibitors of mycobacterial electron transfer flavoprotein oxidoreductase
Source: PLoS One. 2024 Nov 15;19(11):e0312860. doi: 10.1371/journal.pone.0312860 (PMC11567552; doi:10.1371/journal.pone.0312860)

S5 Fig. Residue interactions with SF4 and surrounding binding site. (a) Residues interacting with SF4. (b) Interactions of binding site residues with surrounding residues. Residues are depicted as sticks, while iron and sulfur atoms are represented as balls. The color scheme includes carbon (gray), nitrogen (blue), oxygen (red), sulfur of amino acid residues (yellow), sulfur of SF4 (deep red), iron (magenta), hydrogen bonds (cyan), pi sulfur interactions (dark green), and metal acceptor interactions (black).

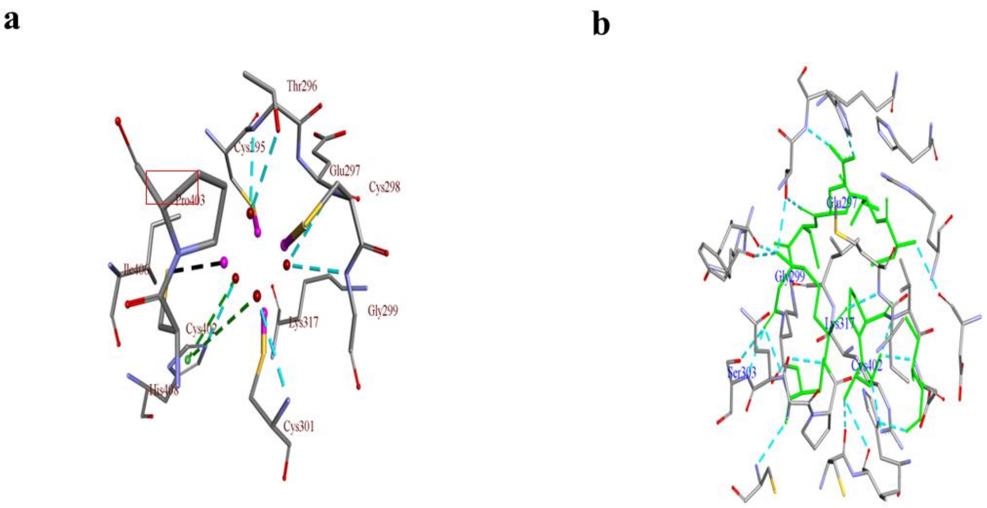

Supplement: S5 Fig — (a) Residues interacting with SF4. (b) Interactions of binding site residues with surrounding residues. Residues are depicted as sticks, while iron and sulfur atoms are represented as balls. The color scheme includes carbon (gray), nitrogen (blue), oxygen (red), sulfur of amino acid residues (yellow), sulfur of SF4 (deep red), iron (magenta), hydrogen bonds (cyan), pi sulfur interactions (dark green), and metal acceptor interactions (black). (PDF) [file pone.0312860.s005.pdf]
