# Supplementary material for: Integrated virtual screening and MD simulation study to discover potential inhibitors of mycobacterial electron transfer flavoprotein oxidoreductase
Source: PLoS One. 2024 Nov 15;19(11):e0312860. doi: 10.1371/journal.pone.0312860 (PMC11567552; doi:10.1371/journal.pone.0312860)
Supplement: S1 Table — (PDF) [file pone.0312860.s008.pdf]

S1 Table. Structure and binding affinity values of top 20 molecules after VS.		
Ligand	2D Structure	Binding Affinity
ZINC000003873157 (Lomefloxacin)	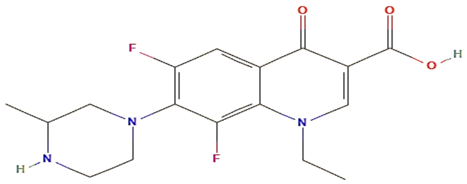	-9.6
ZINC000003812842 (Diacerein)	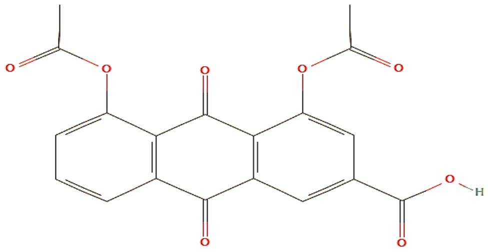	-9.5
ZINC000003812889 (Tibolone)	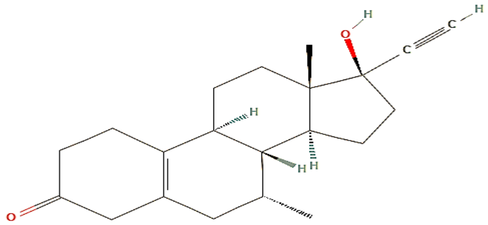	-9.5

ZINC000003794622 (Nadifloxacin)	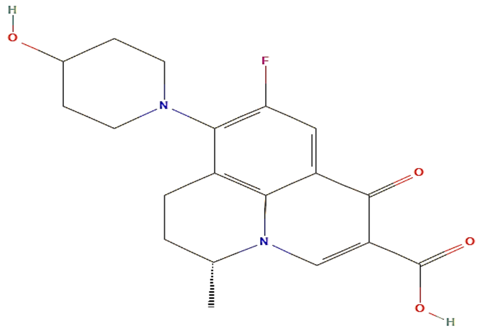	-9.4
ZINC000003812989 (Nalbuphine)	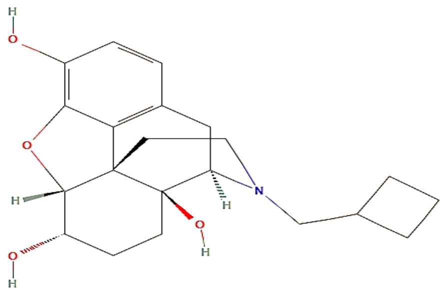	-9.4
ZINC000000603195 (Levonadifloxacin)	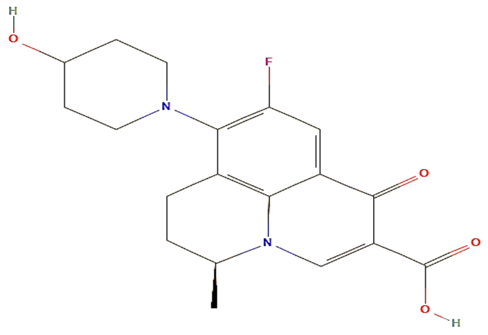	-9.3
ZINC000003607120 (Gatifloxacin)	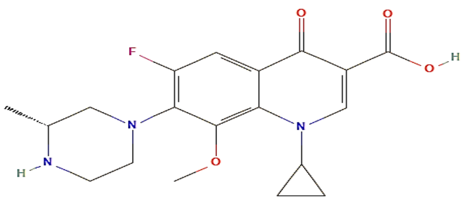	-9.3

ZINC000003919580<br>(Formestane)	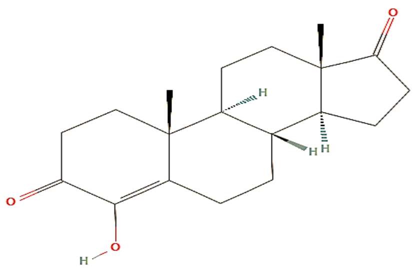	-9.3
ZINC000003875998<br>(Isopregnenone)	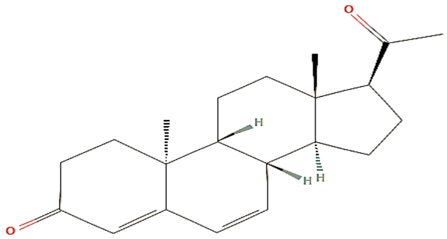	-9.2
ZINC000003812988<br>(Butorphanol)	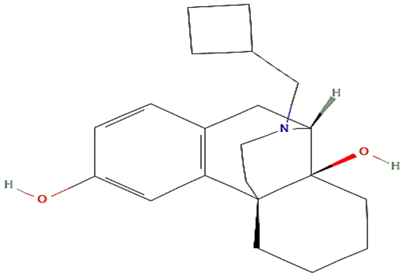	-9.1
ZINC000000601275<br>(Talniflumate)	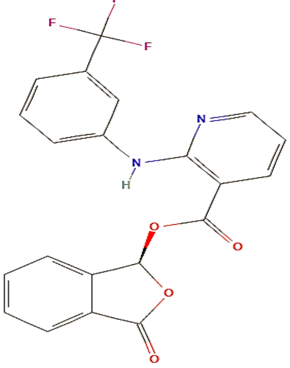	-9.0

ZINC000013509425 (Estrone)	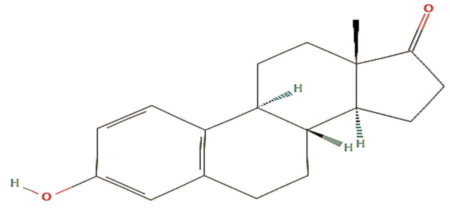	-9.0
ZINC000000001894 (Pefloxacin)	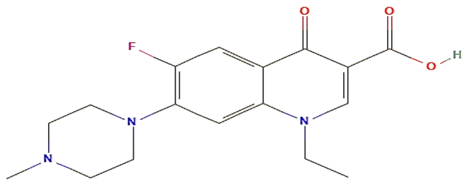	-8.9
ZINC000000020220 (Ciprofloxacin)	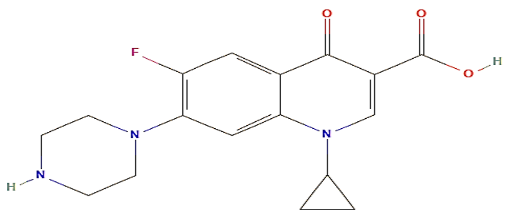	-8.9
ZINC000003779726 (Pazufloxacin)	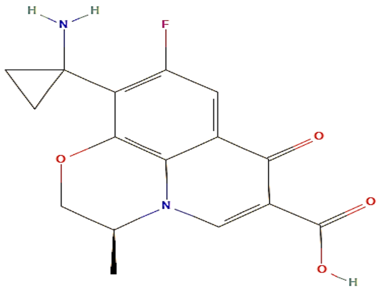	-8.9

ZINC000008143788 (Artemisinin)	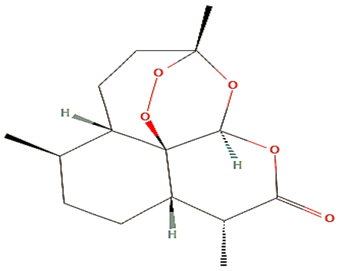	-8.9
ZINC000004081771 (Testolactone)	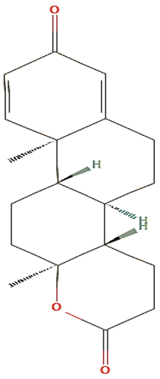	-8.9
ZINC000000000917 (Amifloxacin)	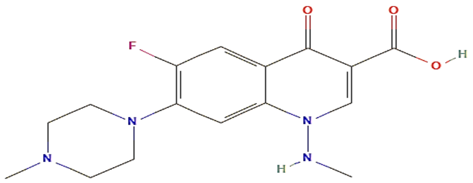	-8.8
ZINC000000119434 (Strychnine)	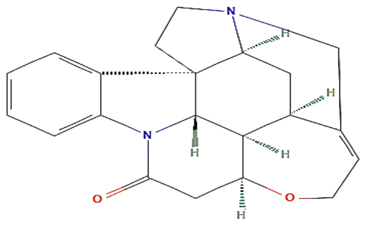	-8.8

ZINC000000538285 (Repirinast)	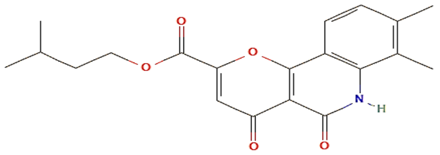	-8.8
